# The Emerging Role of Circular RNAs in Prostate Cancer: A Systematic Review

**DOI:** 10.3389/fcell.2021.681163

**Published:** 2021-07-27

**Authors:** Fan Chao, Shiyu Wang, Cong Zhang, Dunsheng Han, Guoxiong Xu, Gang Chen

**Affiliations:** ^1^Department of Urology, Jinshan Hospital, Fudan University, Shanghai, China; ^2^Research Center for Clinical Medicine, Jinshan Hospital, Fudan University, Shanghai, China

**Keywords:** biomarker, circRNA, metastasis, proliferation, prostate cancer

## Abstract

Prostate cancer is one of the most common malignant tumors that threaten the health of men. It is urgent to explore new molecular targets and develop new drugs for the treatment of prostate cancer. Circular RNAs (circRNAs) are aberrantly expressed in various malignant tumors. The dysregulated circRNAs are involved in the metastasis, tumor growth, drug resistance, and immunosuppression of malignant tumors. The present review systematically summarized publications concerning the biological implications of circRNAs in prostate cancer. The PubMed and Web of Science databases were used to retrieve publications concerning circRNAs and prostate cancer until June 16, 2021. The following keywords were used in the literature search: (circRNA OR circular RNA) AND prostate cancer. 73 publications were enrolled in the present systematic review to summarize the role of circRNAs in prostate cancer. The dysregulated and functional circRNAs were involved in the cell cycle, proliferation, migration, invasion, metastasis, drug resistance and radiosensitivity of prostate cancer. In addition, circRNAs could function through EVs and serve as prognostic and diagnostic biomarkers. Certain circRNAs were correlated with clinicopathological features of prostate cancer. A comprehensive review of the molecular mechanism of the tumorigenesis and progression of prostate cancer may contribute to the development of new therapies of prostate cancer in the future.

## Introduction

Prostate cancer (PCa) is one of the most common malignant tumors that threaten the health of men. The American Cancer Society predicted 248,530 new cases and 69,410 deaths in 2021, ranked 1st and 2nd in men respectively (Siegel et al., 2021). Androgen deprivation therapy (ADT) is the first-line treatment for PCa except for surgery. Nevertheless, castration-resistant PCa (CRPC) is still inevitable for PCa patients (Chandrasekar et al., 2015). Although drugs targeting the androgen receptor (AR) pathway significantly improved the survival of CRPC patients, these drugs did not achieve satisfactory efficacy due to the generation of AR variants and the limitation of drug resistance (Attard and Antonarakis, 2016). Therefore, it is urgent to explore novel molecular targets and develop new drugs for the treatment of PCa.

Circular RNAs (circRNAs) are covalently closed RNAs without 3′ or 5′ ends. High-throughput RNA-sequencing (RNA-seq) is the most commonly used method to identify new circRNAs by detecting the spliced reads that cover the back-splicing junctions. Once generated, circRNAs are highly stable due to their circular structure. Therefore, circRNAs in tissues, blood, or urine can be used as promising biomarkers (Wen et al., 2020). The biological functions of most circRNAs are still unclear. However, studies on certain circRNAs in recent years have discovered that circRNAs may sponge microRNAs (miRNAs), bind to RNA-binding proteins and modulate their activity, regulate transcription or alternative splicing, and be translated to produce novel functional peptides (Kristensen et al., 2019; Xiao et al., 2020).

CircRNAs are aberrantly expressed in various malignant tumors, such as renal cell carcinoma (Wang et al., 2020b), breast cancer (Jahani et al., 2020), and PCa (Vo et al., 2019; Chao et al., 2021b). The dysregulated circRNAs can be involved in the metastasis (Shen et al., 2019), tumor growth (Wu et al., 2020), drug resistance (Zhang et al., 2020a), and immunosuppression (Zhang et al., 2020c) of malignant tumors. Studies in patient-derived xenograft mouse models indicated that intratumor injection of small interference RNA (siRNA) targeting oncogenic circRNA might be a promising treatment of gastric cancer (Zhang et al., 2019). Moreover, circRNAs can serve as promising prognostic or diagnostic biomarkers of malignant tumors (Wang et al., 2018; Rajappa et al., 2020).

The present review systematically summarized publications concerning the biological implications of circRNAs in PCa. A comprehensive review of the molecular mechanism of the tumorigenesis and progression of PCa may contribute to the development of new therapies of PCa in the future.

## Methods

### Search Strategy

The present systematic review was conducted according to the Cochrane guideline. The PubMed and Web of Science databases were used to retrieve publications concerning circRNAs and PCa until June 16, 2021. The following keywords were used in the literature search: (circRNA OR circular RNA) AND prostate cancer. The EndNote X9 (Thompson Reuters, New York, USA) software was used to manage the publications for the present review. In this article, meta-analysis was not performed. The present systematic review was based on previous publications so that ethical consent or approval was not required.

### Inclusion and Exclusion Criteria

Two reviewers (Fan Chao and Shiyu Wang) independently evaluated and selected the publications. Any discrepancy was resolved by the supervisor (Gang Chen). Publications that meet any of the following inclusion criteria were included: (i) expression of the circRNA was quantified in PCa; (ii) biological function and/or mechanism of the circRNA was determined; (iii) prognostic or diagnostic value of the circRNA in PCa was evaluated. Publications that meet any of the following exclusion criteria were excluded: (i) studies that did not meet any of the inclusion criteria; (ii) reviews, books, comments, patents, meeting abstracts and retracted articles; (iii) studies that were not related to circRNA or PCa.

## Results

### Results of the Literature Research

**Figure 1:**
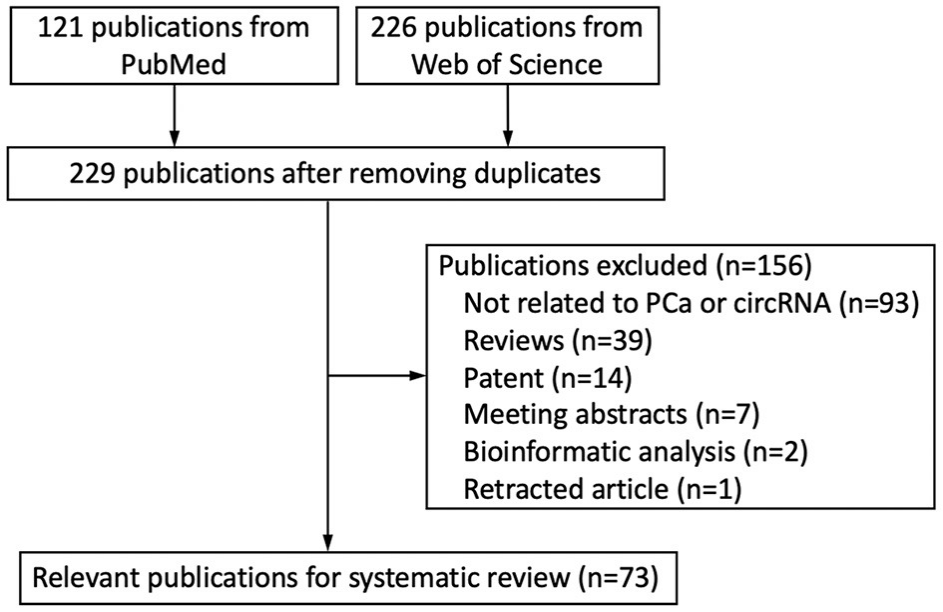
Flow chart of literature research.

We retrieved 347 publications from the databases mentioned above and 229 publications after the removal of duplications. 156 publications were excluded after reviewing. Finally, 73 publications were enrolled in the present systematic review (Figure 1).

### Overview of circRNA

#### History of circRNA Research

In 1976, Sanger et al. discovered that viroids were single-stranded covalently closed circRNA with a highly base-paired rod-like structure. This is the first report on circRNA (Sanger et al., 1976). In 1979, Hsu et al. identified circRNAs in the cytoplasm of eukaryotic cells using electron microscopy (Hsu and Coca-Prados, 1979). In 1993, circular transcripts of the exons of the genomic DNA were identified. However, these circRNAs have been considered to be yielded by mis-splicing of primary transcripts (Cocquerelle et al., 1993). At the same time, *Sry* circRNA was discovered in the testis of adult mice and was considered to be the noise of normal splicing (Capel et al., 1993). Due to the lack of 3′ polyadenylated tails, circRNAs cannot be identified by the classical RNA sequencing which detects the linear RNAs with 3′ polyadenylated tails. Novel RNA sequencing methods which detect RNase R treated RNAs and non-polyadenylated RNAs have been used to identified circRNAs in various species including plants (Lu et al., 2015), fruit flies (Westholm et al., 2014), zebrafish (Shen et al., 2017), mice (Memczak et al., 2013), and human (Salzman et al., 2012). In 2013, Memczak et al. analyzed the biological function of circRNA CDR1as and claimed that circRNAs were a large class of transcripts with the regulatory potential of coding sequences (Memczak et al., 2013).

#### Biogenesis of circRNAs

**Figure 2:**
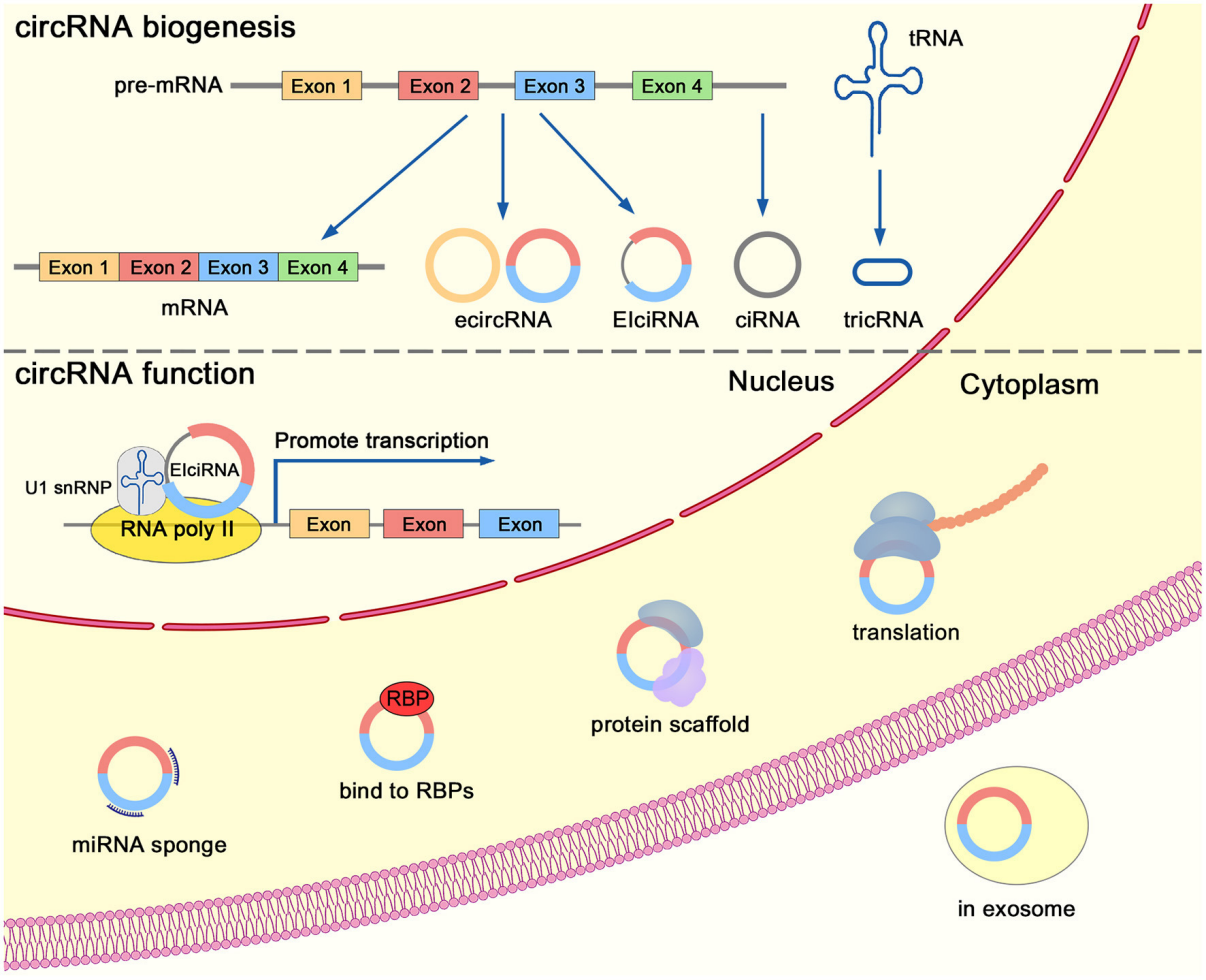
Biogenesis and functions of circRNAs.

Exonic circRNAs are formed by back-splicing of exons and are located in the cytoplasm (Figure 2). Recent studies have revealed that circRNAs are derived from the back-splicing of primary transcripts (Zhang et al., 2020b). Complementary sequences in the introns can mediate the circularization of exons (Zhang et al., 2014). CircRNAs can be generated through an exon-containing lariat precursor in genes that lack intronic complementary sequences (Barrett et al., 2015). Furthermore, the biogenesis of circRNAs can be regulated by RNA-binding proteins in trans. For instance, Quaking (Conn et al., 2015), FUS (Han et al., 2020), and MBNL1 (Ashwal-Fluss et al., 2014) boost the biogenesis of circRNAs, while ADAR1 (Ivanov et al., 2015) and DHX9 (Aktas et al., 2017) suppress the production of circRNAs. HNRNPL (Fei et al., 2017) is a prostate-specific RNA-binding protein that is involved in the formation of circRNAs through back-splicing. Both HNRNPL and its circRNA clients are clinically relevant and aberrantly expressed in PCa (Fei et al., 2017).

#### Category and Nuclear Export of circRNAs

The splicing and circularization of transcripts lead to the generation of various types of circRNAs: exonic circRNAs (ecircRNAs), exon-intron circRNAs (EIciRNAs), intronic circRNAs (ciRNAs), and tRNA intronic circRNAs (tricRNAs) (Figure 2). EcircRNAs are the most investigated class of circRNAs. Although back-splicing of transcripts occurs in the nucleus, a majority of exonic circRNAs were located in the cytoplasm (Chen, 2020). A recent study indicated that DDX39A and DDX39B was involved in the nuclear export of long (> 1,300 nucleotides) and short (< 400 nucleotides) circRNAs, respectively (Huang et al., 2018).

#### Biological Functions of circRNAs

Recent studies have uncovered the biological activity of functional circRNAs (Figure 2). CircRNAs are reported to act as microRNA (miRNA) sponges, to serve as decoys for proteins, to modulate gene expression, to act as scaffolds of proteins, and to function as templates for translation (Chen, 2020). For instance, circRNA *CDR1as* contains more than 70 sponging targets for miRNA *miR-7*. Although *CDR1as* is resistant to the miRNA-mediated degradation of target RNAs, it strongly inhibits the activity of *miR-7* and thereby increases the level of other *miR-7* targets (Hansen et al., 2013). In addition, *CDR1as* can suppress the metastasis of melanoma through modulating the activity of its interactor, IGF2BP3 (Hanniford et al., 2020). *CircSMARCA5* interacts with its host gene through regulating transcription of partial exons (Xu et al., 2020c). *CircMALAT1* can serve as a brake to retard *PAX5* mRNA translation in ribosomes (Chen et al., 2020b). *Circ-Ccnb1* acts as a protein scaffold of Ccnb1 and Cdk1, resulting in the dissociation of cyclin B1-Cdk1 complex and cell death (Fang et al., 2019). Several studies have reported that circRNAs could encode peptides. For instance, *circFNDC3B* encodes a novel protein and inhibits tumor progression in colon cancer (Pan et al., 2020).

### The role of circRNAs in PCa

**Figure 3:**
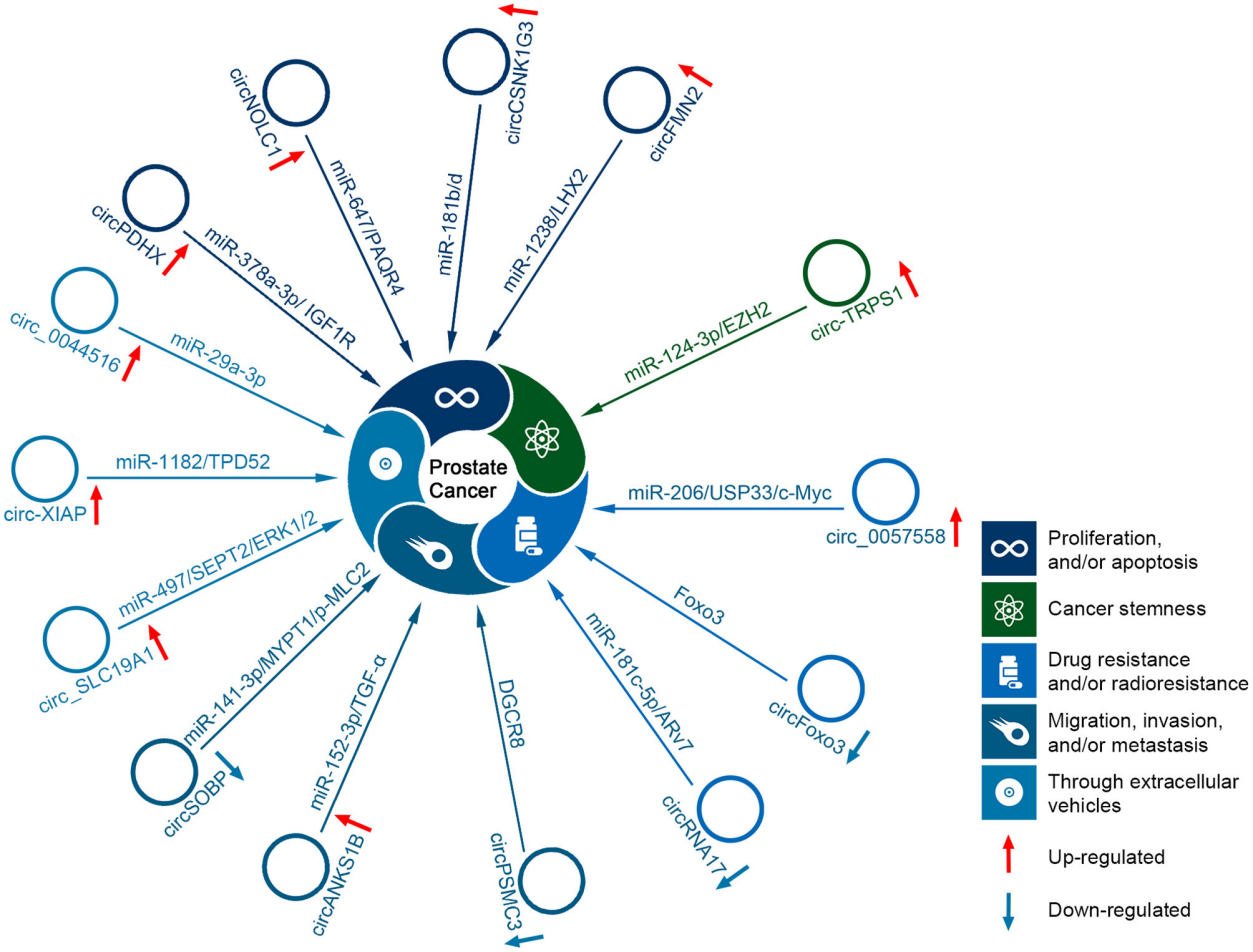
Schematic illustration of the representative functional circRNAs.

The functions and downstream targets of the dysregulated circRNAs in PCa are listed in Table 1. The dysregulated and functional circRNAs are involved in the cell cycle, proliferation, migration, invasion, metastasis, drug resistance and radiosensitivity of PCa (Figure 3). Some circRNAs can serve as prognostic and diagnostic biomarkers. Here, we summarized the role of circRNAs in PCa.

**Table 1:** Overview of functional circRNAs in prostate cancer.

**CircRNA**	**circBase ID**	**Expres-sion in PCa**	**Function**	**Upstream regulator**	**Downstream target**	**Clinical relevance**	**Reference**
**Regulating cell cycle, proliferation, and apoptosis**							
circ_0004417	hsa_circ_0004417	Low	Inhibited proliferation and invasion	NR	miR-1228, p-Akt and E-cadherin	NR	Xia et al., 2021
hsa_circ_0062019	hsa_circ_0062019	High	Promoted proliferation, invasion and migration	NR	miR-195-5p/HMGA2	NR	Wang et al., 2021b
circSLC8A1	hsa_circ_0000994	Low	Inhibited proliferation, migration and invasion	NR	miR-21, MAPK signaling pathway, and chemokine signaling pathway	Survival	Wang et al., 2021a
CDR1as	hsa_circ_0001946	High	Promoted proliferation, invasion and migration	NR	miR-641/XIAP	NR	Niu et al., 2021
circUBAP2	hsa_circ_0001846	High	Promoted proliferation	NR	miR-1244/MAP3K2	NR	Li et al., 2021b
circRNA_100395	hsa_circ_0015278	Low	Inhibited proliferation, migration, invasion and EMT, altered cell cycle distribution	NR	miR-1228	Tumor size, Gleason score, tumor stage, and lymph node metastasis	He et al., 2021
circPDHX	hsa_circ_0003768	High	Promoted proliferation, tumor growth, invasion, colony formation	NR	miR-378a-3p/ IGF1R	Gleason score, T stage and survival	Mao et al., 2020
circ_0062020	NR	High	Promoted proliferation, tumor growth, migration, and invasion; inhibited radiosensitivity and apoptosis	NR	miR-615-5p/TRIP13	Gleason score, tumor size, TNM stage	Li et al., 2020a
circGOLPH3	hsa circ 0072068	High	Promoted proliferation; inhibited apoptosis	NR	CBX7	NR	Gong et al., 2020
circCDK13	hsa_circ_0079929	NR	Promoted tumorigenesis, proliferation	E2F5/ CDK13	miR-212-5p/miR-449a and E2F5	NR	Qi et al., 2021
circNOLC1	hsa_circ_0000257	High	Promoted proliferation, migration	NR	miR-647/PAQR4	NR	Chen et al., 2020c
circ_KATNAL1	hsa_circ_0008068	Low	Inhibited proliferation, invasion, migration	NR	miR-145-3p/WISP1	NR	Zheng et al., 2020a
circ_0057553	hsa_circ_0057553	High	Promoted viability, migration, invasion, glycolysis; inhibited apoptosis	NR	miR-515-5p/YES1	NR	Zhang et al., 2020e
hsa_circ_0007494	hsa_circ_0007494	Low	Inhibited proliferation, invasion, tumor growth	NR	miR-616/PTEN	NR	Zhang et al., 2020d
circMBOAT2	hsa_circ_0007334	High	Promoted proliferation, migration, invasion, tumorigenesis, metastasis	NR	miR-1271-5p/mTOR	Gleason score, T stage, prognosis	Shi et al., 2020
circFOXO3	hsa_circ_0006404	High	Promoted viability, metastasis, cell cycle, proliferation; inhibited apoptosis	NR	miR-1299/CFL2, miR-29a-3p/SLC25A15	Gleason score	Kong et al., 2020; Li et al., 2021a
circFMN2	hsa_circ_0005100	High	Promoted cell growth	NR	miR-1238/LHX2	T stage, lymph node metastasis, distant metastasis	Shan et al., 2020
circCRKL	hsa_circ_0001206	High	Promoted cell cycle, migration, invasion, tumor growth; inhibited apoptosis	DHX9	miR-141/KLF5, miR-1285-5p/SMAD4	T stage, Gleason score	Song et al., 2019; Nan et al., 2020
circHIPK3	hsa_circ_0000284	High	Promoted viability, proliferation, migration, invasion, tumor growth; inhibited apoptosis	NR	miR-338-3p/Cdc25B/Cdc2, miR-193a-3p/MCL1, miRNA-338-3p/ADAM17	Gleason score, T stage	Cai et al., 2019; Chen et al., 2019a; Liu et al., 2020
circDDX17	hsa_circ_0002211	Low	Inhibited mobility, proliferation, invasion	NR	miR-346/LHPP	NR	Lin et al., 2020
cir-ITCH	hsa_circ_ 0001141	Low	Inhibited proliferation, migration, invasion	NR	miR-17	NR	Li et al., 2020c
circ-0016068	hsa_circ_0016068	High	Promoted tumor growth, metastasis, EMT	NR	miR-330-3p/BMI-1	Survival	Li et al., 2020b
circZMIZ1	hsa_circ_0005844	High	Promoted proliferation, cell cycle	NR	AR, AR-V7	NR	Jiang et al., 2020
circ-MTO1	NR	Low	Inhibited proliferation, invasion	NR	miR-17-5p	Survival, T stage, N stage	Hu and Guo, 2020
circSMARCA5	hsa_circ_0001445	High	Promoted proliferation, metastasis, glycolysis, cell cycle	Androgen	miR-432/PDCD10	NR	Kong et al., 2017; Dong et al., 2020a
circ_0088233	hsa_circ_0088233	High	Promoted proliferation, migration, invasion, cell cycle, apoptosis	NR	miR-185-3p	TNM stage	Deng et al., 2020
circABCC4	hsa_circ_0030586	High	Promoted proliferation, cell cycle, migration, invasion, tumor growth	NR	miR-1182/FOXP4	Survival	Huang et al., 2019a
circSMAD2	hsa_circ_0000847	Low	Inhibited proliferation, migration, EMT	NR	miR-9/STAT3	NR	Han et al., 2019
circRNA0005276	hsa_circ_0005276	High	Promoted proliferation, migration, EMT	NR	FUS/XIAP	NR	Feng et al., 2019
circCSNK1G3	hsa_circ_0001522	High	Promoted proliferation	NR	miR-181b/d	NR	Chen et al., 2019b
circ-ITCH	hsa_circ_0001141	Low	Inhibited proliferation, tumor growth, colony formation; promoted apoptosis	NR	miR-197, miR-17-5p/HOXB13	Survival, PSA level, Gleason score, T stage, lymph node metastasis, survival	Huang et al., 2019b; Wang et al., 2019; Yuan et al., 2019
circAMOTL1L	hsa_circ_0000350	Low	Inhibited migration, invasion, cell growth, EMT	p53/RBM25	miR-193a-5p/Pcdha	Gleason score	Yang et al., 2019
circMYLK	hsa_circ_0141940	High	Promoted proliferation, invasion, migration; inhibited apoptosis	NR	miR-29a	NR	Dai et al., 2018
**Specifically governing migration and invasion**							
circSOBP	hsa_circ_0001633	Low	Inhibited migration, invasion, metastasis, amoeboid migration	NR	miR-141-3p/MYPT1/p-MLC2	Gleason score, and grade group	Chao et al., 2021a
circANKS1B	hsa_circ_0007294	High	Promoted migration and invasion	NR	miR-152-3p/TGF-α	Survival, Gleason score, T stage, lymph node metastasis	Tao et al., 2021
circRNA-ARC1	hsa_circ_0090923	NR	Promoted migration, invasion, and metastasis	AR	miR-125b-2-3p or miR-4736/PPARγ/MMP-9	NR	Deng et al., 2021
hsa_circ_0001165	hsa_circ_0001165	NR	Promoted EMT	NR	hsa-miR-187-3p/TNF	NR	Yan et al., 2020
hsa_circ_0001085	hsa_circ_0001085	NR	Promoted EMT	NR	hsa-miR-196b-5p/TGF-β pathway, hsa-miR-451a/MAPK pathway	NR	Yan et al., 2020
circRNA-51217	NR	NR	Promoted invasion, metastasis	R-2HG	miRNA-646/TGFβ1/p-Smad2/3	NR	Xu et al., 2020a
circular RNA_LARP4	NR	Low	Inhibitd migration, invasion	NR	FOXO3A	prognosis	Weng et al., 2020
circPSMC3	hsa_circ_0021977	Low	Inhibited migration, invasion	NR	DGCR8	NR	Dong et al., 2020b
circ-102004	NR	High	Promoted migration, invasion	NR	ERK, JNK and Hedgehog pathways	NR	Si-Tu et al., 2019
**Involving in the drug resistance and radiosensitivity**							
circ_0057558	hsa_circ_0057558	High	Promoted proliferation and colony formation, inhibited cell cycle arrest and sensitivity to docetaxel	NR	miR-206/USP33/c-Myc	NR	Ding et al., 2021
circFoxo3	hsa_circ_0006404	Low	Inhibited cell survival, migration, invasion, EMT; promoted chemoresistance to docetaxel	NR	Foxo3/EMT	Grade, survival	Shen et al., 2020
hsa_circ_0000735	hsa_circ_0000735	High	Promoted viability, colony formation, cell cycle, tumor growth; inhibited docetaxel sensitivity	NR	miR-7	Survival	Gao et al., 2020
circ_CCNB2	hsa_circ_0035483	High	Inhibited radiosensitivity and apoptosis; promoted colony formation, migration, invasion	NR	miR-30b-5p/KIF18A	NR	Cai et al., 2020
circUCK2	hsa_circ_001357	Low	Inhibited enzalutamide resistance, proliferation, invasion	NR	miR-767-5p/TET1	NR	Xiang et al., 2019
circRNA17	hsa_circ_0001427	Low	Inhibited enzalutamide resistance, invasion	NR	miR-181c-5p/ARv7	Gleason score	Wu et al., 2019
circZNF609	hsa_circ_0000615	High	Promoted colony formation, viability, migration, invasion, metastasis, glycolysis; Inhibited apoptosis, radiosensitivity,	NR	miR-186-5p/YAP1/AMPK pathways, miR-501-3p/HK2	NR	Jin et al., 2019; Du et al., 2020
**Regulating cancer stemness**							
circ-TRPS1	hsa_circ_ 0006950	High	Promoted proliferation, metastasis, stemness	NR	miR-124-3p/EZH2	T stage, N stage, M stage	Sha et al., 2020
**Functioning through EVs**							
circ_0044516	hsa_circ_0044516	High	Through exosoms; promoted proliferation, migration, invasion	NR	miR-29a-3p	NR	Li et al., 2020d
circ-XIAP	hsa_circ_0005276	High	Promoted docetaxel resistance, proliferation, migration and invasion; inhibited cell cycle arrest and apoptosis; through exosomes	NR	miR-1182/TPD52	NR	Zhang et al., 2021
circ_SLC19A1	hsa_circ_0062019	High	Promoted proliferation, invasion, through EVs	NR	miR-497/SEPT2/ERK1/2	NR	Zheng et al., 2020b

*EMT, epithelial-mesenchymal transition; EVs, extracellular vesicles; NR, not reported*.

#### Regulating Cell Cycle, Proliferation, and Apoptosis

The most basic characteristics of cancer cells involve their long-lasting and long-term proliferation ability. Normal tissues can precisely control the generation and release of growth-promoting signals, which govern the start and progress of the cell proliferation-differentiation cycle. This maintains the balance of cell number, thereby ensuring the structure and function of normal tissues. These signals in cancer cells are dysregulated, thereby they can sustain the proliferation of cancer cells (Hanahan and Weinberg, 2011). In addition, tumor cells have evolved a series of mechanisms that limit or allow them to avoid apoptosis. Under pathological conditions, especially in cancer, cells lose the ability to undergo cell death induced by apoptosis, leading to uncontrolled proliferation. There are several mechanisms by which cells escape programmed cell death, one of which is the expression of anti-apoptotic molecules (Mohammad et al., 2015).

Current studies have revealed that circRNAs were involved in the proliferation, cell cycle, and apoptosis of PCa cells. For instance, Shan et al. reported that knockdown of *circFMN2* suppressed tumor growth of PCa *in vivo* and inhibited proliferation of PCa cells by inducing cell-cycle arrest and apoptosis through regulating *miR-1238*/*LHX2* axis (Shan et al., 2020). Mao et al. reported that *circPDHX* was associated with Gleason score, pathological T stage and overall survival of PCa, promoted cell proliferation *in vitro* and tumor growth *in vivo* (Mao et al., 2020). Liu et al. suggested that *circHIPK3* expression was upregulated in PCa and promoted G2/M transition by sponging *miR-338-3p* (Liu et al., 2020). Deng et al. claimed that the suppression of *circ_0088233* reduced cell proliferation and induced G1 phase arrest and apoptosis through targeting *hsa-miR-185-3p* (Deng et al., 2020). Zhang et al. indicated that the knockdown of *circ_0057553* could inhibit cell viability and facilitate apoptosis (Zhang et al., 2020e). Huang et al. suggested that knockdown of *circABCC4* inhibited tumor growth *in vivo* and cell-cycle progression *in vitro* by targeting *miR-1182-FOXP4* regulatory axis (Huang et al., 2019a).

#### Governing Migration and Invasion

The mechanism of cancer metastasis was largely an enigma until 2000. The multiple steps of invasion and metastasis have been schematized as a series of independent steps, commonly termed as the invasion-metastasis cascade (Fidler, 2003; Talmadge and Fidler, 2010). This description depicted a series of cellular biological changes. Invasion and metastasis of cancer cells start with local invasion, then intravasation of the cancer cells allows them to infiltrate the surrounding blood vessels and lymph vessels. Subsequently, cancer cells are transported through the lymphatic or blood system, followed by the extravasation which helps cancer cells to escape from the vessels into the parenchyma of the distal tissues. Escaped cancer cells grow into small nodules, which are termed micrometastases. The last step, colonization, refers to the growth of micrometastases into metastatic tumors. Research on invasion and metastasis capabilities has been greatly accelerated in recent years.

Epithelial-mesenchymal transition (EMT) plays an important role in the metastasis of malignant tumors (Huber et al., 2005). CircRNAs are involved in the EMT program. Yan et al. identified EMT-related circRNAs through RNA-seq in interferon-γ (IFN-γ) induced EMT cells and found that *hsa_circ_0001165* and *hsa_circ_0001085* were EMT-related circRNAs (Yan et al., 2020). *Hsa_circ_0001165* and *hsa_circ_0001085* played a regulatory role in the EMT of PCa. Feng et al. reported that *circ0005276* promoted cell proliferation and EMT through interacting with *FUS* (Feng et al., 2019). Han et al. found a decrement of *circSMAD2* in PCa tissues. Restoration of *circSMAD2* could inhibit the impaired EMT process through *miR-9* inhibition (Han et al., 2019). Yang et al. claimed that *p53* regulated EMT through *circAMOTL1L/miR-193a-5p/Pcdha* regulatory axis (Yang et al., 2019). Li et al. suggested that *circ-0016068* promoted EMT of PCa cells by regulating the *miR-330-3p/BMI-1* axis (Li et al., 2020b). Shen et al. reported that *circFoxo3* inhibited PCa cell migration and invasion through regulating *Foxo3* and EMT (Shen et al., 2020).

In addition to EMT, circRNAs can govern the metastasis of PCa through other pathways. Xu et al. found that *circRNA-51217* could sponge *miRNA-646*, which could induce TGFβ1/p-Smad2/3 signaling pathway and promote PCa cell invasion (Xu et al., 2020a). Weng et al. suggested that *circular RNA_LARP4* could inhibit cell migration and invasion through upregulating *FOXO3A* (Weng et al., 2020). Si-Tu et al. reported that *circ-102004* played an oncogenic role by promoting migration and invasion of PCa cells (Si-Tu et al., 2019). Overexpression of *circ-102004* alters *ERK, JNK*, and Hedgehog pathways. Chao et al. found that *circSOBP* inhibited amoeboid migration and metastasis of PCa cells through *miR-141-3p/MYPT1/p-MLC2* axis (Chao et al., 2021a).

#### Involving in the Drug Resistance and Radiosensitivity

In 1940, Charles Huggins reported that castration of men with PCa induced dramatic symptomatic improvements and resulted in the regression of cancer sites (Huggins and Hodges, 1941). Since then, ADT has been a mainstay therapy for advanced PCa, including medical castration, surgical castration, and inhibitors for androgen biosynthesis (Feng and He, 2019). Given psychological and aesthetic concerns, medical castration has been more adopted instead of surgical castration in the treatment for PCa. However, progression to castration-resistant PCa (CRPC) is inevitable after ADT. Novel second-generation antiandrogens have been developed to achieve better blockade for androgen (Tran et al., 2009), including darolutamide, apalutamide, and enzalutamide. The second-generation antiandrogens have successfully prolonged the survival of PCa patients. Unfortunately, the prolonged survival is temporary. The CRPC can be resistant to the latest antiandrogens.

CircRNAs are involved in the drug resistance of various cancers (Xu et al., 2020b). Recent studies have investigated the role of circRNAs in the CRPC. Cao et al. (2019) identified 13 circRNAs derived from the *AR* gene through RNA-seq of 47 metastatic CRPC samples, cell models, and RNase R RNA-seq of patient-derived xenografts (PDXs). Expression of the four most abundant circRNAs are upregulated during the castration-resistant progression of PDXs and can be detected in the plasma of patients with PCa. These AR-derived circRNAs might serve as biomarkers for CRPC. Greene et al. (2019) uncovered that circRNAs were more often downregulated in enzalutamide-resistant PCa cells using a high-throughput circRNA microarray. *Hsa_circ_0004870* was one of the downregulated circRNAs which might mediate the development of enzalutamide resistance in PCa. Wu et al. (2019) revealed the low expression of *circRNA17* in CRPC C4-2 enzalutamide-resistant cell lines compared to the parental sensitive cells. This work suggested that *circRNA17* could govern the enzalutamide sensitivity and cell invasion through the *miR-181c-5p*/*ARv7* axis. Xiang et al. (2019) identified that *circUCK2* was also downregulated in enzalutamide-resistant cells. Targeting these circRNAs might help develop new therapies for the treatment of CRPC.

Docetaxel is the first chemotherapeutic agent which was proved to prolong the survival of patients with metastatic CRPC (Sartor and de Bono, 2018). Results of the STAMPEDE trial advocated the upfront use of docetaxel for patients with metastatic hormone-naive PCa (Clarke et al., 2019). Recent studies have revealed that circRNAs were involved in the docetaxel sensitivity of PCa. Shen et al. (2020) suggested that reduction *circFoxo3* boosted chemoresistance to docetaxel of PCa. Depletion of *circFoxo3* using siRNAs promoted chemoresistance to docetaxel in mice with xenografts, while the delivery of *circFoxo3* prolonged the survival of tumor-bearing mice and enhanced the sensitivity to docetaxel. Gao et al. (2020) uncovered that *hsa_circ_0000735* was upregulated in docetaxel-resistant PCa tissues and was correlated with worse overall survival. Knockdown of *hsa_circ_0000735* boosted sensitivity to docetaxel of PCa cells and inhibited viability *in vivo*. In addition, suppressing *hsa_circ_0000735* promoted docetaxel sensitivity and suppressed tumor growth *in vivo*. Zhang et al. reported that exosomal *circ-XIAP* promoted docetaxel resistance in PCa by altering *miR-1182/TPD52* axis (Zhang et al., 2021).

External radiotherapy is a radical treatment for low-risk PCa, which has been considered to achieve the same effect as surgical treatment. Resistance to radiotherapy is an obstacle for the treatment of PCa. Du et al. (2020) reported that *circ-ZNF609* suppressed the radiosensitivity of PCa. Knockdown of c*irc-ZNF609* inhibited the radioresistance, viability and promoted apoptosis through regulating glycolysis. Silencing *circ-ZNF609* enhanced the sensitivity to radiotherapy *in vivo*. Cai et al. (2020) focused on the effect of *circ_CCNB2* on radiosensitivity of PCa. Depletion of *circ_CCNB2* facilitated the radiosensitivity of irradiation-resistant PCa cells *in vitro* and *in vivo* through suppressing autophagy via *miR-30b-5p/KIF18A* axis.

#### Regulating Cancer Stemness

Cancer is characterized by infinite proliferation of the malignant cells with different morphology and functions. There are currently two models that explain this cellular diversity in tumors. The first model that explain the initiation and development of cancer postulates that each sequential accumulation of mutations promotes the loss of specific tissue characteristics, until the occurrence of dedifferentiation and the regression into a more primitive phenotype. In this model, every cancer cell has a similar tumorigenic potential. The second mode is the cancer stem cell (CSC) hypothesis (Aguilar-Gallardo and Simon, 2013; Pattabiraman and Weinberg, 2014). The CSCs refer to cells in tumors that have the ability to self-renew and produce heterogeneous cancer cells. These cells with stemness are responsible for producing various offspring of highly proliferative cells that form the bulk of the tumors.

So far, only one study reported the role of circRNA in regulating the stemness of PCa cells. Sha et al. (2020) indicated that *circ-TRPS1* was upregulated in high-grade PCa tissues and was associated with aggressive PCa phenotypes. Knockdown of *circ-TRPS1* inhibited proliferation and metastasis of PCa through *miR-124-3p/EZH2* axis-mediated stemness.

#### Functioning Through Extracellular Vesicles (EVs)

There are two types of EVs based on their size and origin: exosomes and microvesicles (van Niel et al., 2018). Exosomes ranged in size from 50 to 100 nm are derived from intraluminal vesicles (ILVs) during the formation of multivesicular endosomes (MVEs), in which ILVs are formed by the inward budding of an endosomal membrane of MVEs and then secreted upon the fusion of MVE membrane and the cell membrane. Microvesicles ranged in size from 50 to 1,000 nm in diameter are also called oncosomes due to their role in cellular communication in cancer (Al-Nedawi et al., 2008). Microvesicles are directly generated from the outward budding followed by fission of the cell membrane. Subsequently, microvesicles are released into the extracellular space.

EVs were usually isolated from body fluids or cell culture media using special kits or ultra-high-speed centrifugation, followed by identification using an electron microscope. CircRNAs in blood or EVs can be served as biomarkers or functional factors (Hu et al., 2020; Wen et al., 2020). *CircSLC19A* (Zheng et al., 2020b) was increased in both PCa cells and their EVs. EVs with high *circSLC19A* could be taken up by PCa cells and boosted cell proliferation and invasion. Exosomal *circSLC19A* promoted proliferation and invasion of cells through the *miR-497/septin 2* axis. Li et al. (2020d) identified the existence of *circ_0044516* in exosomes using circRNA microarray. *Circ_0044516* was upregulated in the exosomes of PCa patients and cell lines. However, this study did not investigate whether exosomal circRNAs could be taken up by cells and function. Zhang et al. discovered that *circ-XIAP* was up-regulated in exosomes from docetaxel-resistant cell lines and could be transmitted via exosomes (Zhang et al., 2021). Adding exosomes from docetaxel-resistant cells increased *circ-XIAP* level in prostate cancer cell lines, suggesting that exosomes containing *circ-XIAP* could be absorbed.

#### Serving as Diagnostic and Prognostic Biomarkers

The characteristics of circRNAs make them promising biomarkers. Firstly, circRNAs are specifically expressed in various tissues and body fluids. CircRNAs can be enriched in exosomes and released from their original tissues into various body fluids, including plasma, saliva, and urine. Moreover, they can be released from dead or dying cells with the rupture of cell membranes. Secondly, the covalently closed structure and resistance to RNase have endowed circRNAs with high stability. The half-life of circRNAs is about 2.5 times longer than linear RNAs in cells and 6.3 times longer in exosomes (Jeck et al., 2013; Li et al., 2015; Enuka et al., 2016). Thirdly, circRNAs can be easily measured using RNA-seq or quantitative polymerase chain reaction (qPCR). The exact copy number and mutant of circRNAs can be detected. This is an advantage when comparing with protein biomarkers which are quantified by antigen-antibody interaction. A current meta-analysis suggested that *CDR1as* was a reliable prognostic and diagnostic biomarker for solid tumors by summarizing 26 studies (Zou et al., 2020). CircRNAs can serve as remarkable biomarkers in colorectal cancer (Yuan et al., 2020), lung cancer (Yang et al., 2020), gastric cancer (Chen et al., 2020a), and glioma (Ding et al., 2020).

Current studies indicated that circRNAs were correlated with clinicopathological features of PCa. The expression levels of *circMBOAT2* (Shi et al., 2020), *circFoxo3* (Shen et al., 2020), *circCRKL* (Song et al., 2019; Nan et al., 2020), and *circHIPK3* (Cai et al., 2019; Liu et al., 2020) were associated with the histological grade of PCa. The expression levels of *circFoxo3* (Shen et al., 2020), *circ-0016068* (Li et al., 2020b), *circ-MTO1* (Hu and Guo, 2020), *hsa_circ_0000735* (Gao et al., 2020), *circ-ITCH* (Wang et al., 2019), and *circABCC4* (Huang et al., 2019a) were associated with the survival of patients with PCa, suggesting that circRNAs had the potential to predict the prognosis of PCa patients. Wang et al. established an eight-circRNA prognosis model to predict the biochemical recurrence of PCa. This prognosis model based on circRNAs was better than the clinical indexes (Wang et al., 2020a).

## Discussion

CircRNAs has attracted more and more attention from the scientific community, and their involvement in the molecular regulation in cells has been continuously revealed. Similar to protein and lncRNAs, circRNAs can regulate the malignant behavior of cancer cells through cell signaling pathways. Although a large number of circRNAs have been discovered and even included in the databases, the biological implications of most circRNAs in eukaryotic cells remained unclear. In recent years, a large number of studies have systematically identified circRNAs and their functions. Targeting circRNAs has great potential in the field of cancer intervention, diagnosis and treatment. In the present review, we systematically summarized the functional circRNAs in PCa. Various circRNAs governed the proliferation, apoptosis, migration, invasion, drug resistance, and radiosensitivity in PCa. In addition, circRNAs could function through EVs and serve as prognostic and diagnostic biomarkers.

The limitations and pitfalls of the studies on circRNAs should not be ignored. The studies concerning circRNAs are in the preliminary stage. At the same time, the application of circRNAs in clinical practice remained up in the air. Current studies reported the dysregulation of circRNAs in PCa. These circRNAs that were clinically significantly dysregulated could serve as remarkable biomarkers. A prognosis model based on multiple circRNAs has been established to predict the biochemical recurrence of PCa (Wang et al., 2020a). However, circRNA biomarkers were not used in clinical practice due to the lack of further research. Subsequent clinical researches should be carried out to verify the sensitivity and specificity of circRNA biomarkers according to larger cohorts.

Although circRNAs are more stable than linear RNAs, they are still not as good as proteins when serving as biomarkers. The expression abundance of circRNAs in cells is low and is lower in body fluids, so it is very difficult to quantitatively detect them using traditional methods. Thus, it is urgent to develop new detecting techniques in the future. In addition, detecting techniques and bioinformatic analyses should be standardized to collect reliable data. When these technical obstacles are solved in the future, circRNAs will play an important role in the field of biomarkers and are expected to replace existing markers.

Although biological functions of certain circRNAs have been revealed, the application based on the functional circRNAs in the treatment of PCa is poorly studied. The precise delivery of functional circRNAs to the cancer cells, as well as the overexpression or suppression of functional circRNA in the cancer cells, are the current difficulty in the clinical use of circRNAs. The delivery can be achieved using nanoparticles or viral vectors; however, their specificity is not satisfactory. Since circRNAs are functional in human cells, non-selective application to the human body may cause a variety of side effects, so accurate delivery is very important. Recombinant adeno-associated virus (rAAV) vectors can be used in gene therapy of cancer due to its advantages including tissue specificity, long-term transgene expression, and low immunogenicity (Luo et al., 2015; Santiago-Ortiz and Schaffer, 2016). Sun et al. developed a prostate-specific rAAV that inhibited tumor growth of PCa through gene silencing (Sun et al., 2010). Ai et al. investigated the transgene efficiency of various serotypes of rAAVs and discovered that rAAV6.2 and rAAV7 outperformed other serotypes in the whole prostate (Ai et al., 2016). Therefore, the delivery of circRNAs or their siRNAs through prostate-specific rAAVs could be used as novel gene therapy for PCa in the future.

Although a number of studies have reported the biological functions of circRNAs in prostate cancer, it is uncertain which ones are prostate-specific due to the lack of experimental verification. Further research should focus on discovering the prostate-specific circRNAs. Targeting prostate-specific circRNA and developing new therapeutic targets are of great significance.

Researchers have reported the dysregulation of circRNAs and their biological function as well as the mechanism. Nevertheless, the reason why circRNAs were dysregulated was poorly studied. A previous study reported that the expression level of circRNA was positively correlated with its host gene (Chao et al., 2021a). Since circRNA and mRNA derived from the same gene are encoded by the same genomic sequence, they may be regulated by common factors. If a certain circRNA has the same biological functions as the protein encoded by its host gene, targeting their upstream regulatory mechanism may achieve a better effect. Nevertheless, Yu et al. found that the expression trend of circRNA was opposite to its host gene, and might be governed by the RNA-binding proteins that mediated the cyclization of circRNAs (Yu et al., 2018). Targeting the upstream regulators of circRNAs is expected to fundamentally block or promote their biological functions, thereby exerting a therapeutic effect on human diseases. Hence, the regulators of circRNAs should be systematically determined in the future.

## Data Availability Statement

The original contributions presented in the study are included in the article/supplementary material, further inquiries can be directed to the corresponding author.

## Author Contributions

FC performed the literature search, analyzed the data, made the schematic diagram, and wrote the manuscript. SW performed the literature search and the review on the overview of circRNA. CZ performed the review on the role of circRNA in PCa. DH determined the inclusion and exclusion criteria. GX revised the manuscript. GC conceived the idea and revised the manuscript. All authors contributed to the article and approved the submitted version.

## Conflict of Interest

The authors declare that the research was conducted in the absence of any commercial or financial relationships that could be construed as a potential conflict of interest.

## Publisher's Note

All claims expressed in this article are solely those of the authors and do not necessarily represent those of their affiliated organizations, or those of the publisher, the editors and the reviewers. Any product that may be evaluated in this article, or claim that may be made by its manufacturer, is not guaranteed or endorsed by the publisher.
